# Some Observations in Surgery, Illustrated by Cases and Plates

**Published:** 1816-12

**Authors:** 


### PART II.

### COMPREHENSIVE ANALYTICAL REVIEW OF MEDICAL LITERATURE.

"Tros, tyriusve, nobis nullo discrimine agetur."

*Some Observations in Surgery, illustrated by Cases and Plates.* By A. Copland Hutchison, M.D. late Surgeon of Deal Hospital. London, 1810, Octavo, pp. 168.

That experience gained amid the roar of cannon, the clash of bayonets, and the conflicts of the elements, is now shedding its salutary influence over the mansions of peace, and the abodes of disease. This is "ex *fumo* dare lucem;" and Dr. Hutchison has, in the volume before us, contributed his mite towards the general good. He has not, indeed, issued a costly volume of hot-pressed speculations, but a moderate-priced book of practical facts. As such, we deem it our duty to recommend it to the library of every surgeon who is not above receiving information from the experience of others, and who does not believe that all knowledge is concentrated in himself.

This work treats of amputation; erysipelatous inflammation; aneurism of the popliteal artery; on the use of the probe in operations for punctured arteries; on necrosis; the history of an extraordinary case of abscess of the liver; on lumbar abscess; on un-united fractures: besides a great body of interwoven facts, observations, criticisms, and suggestions. We shall endeavour to present our readers with some valuable specimens from all, or most of these different subjects.

*Amputation.*—In our review of Mr. Guthrie's work, we took an opportunity of differing in sentiment from that gentleman, respecting the *time* for operating after the receipt of the injury.

"We have seen (said we) many instances, where the operation was performed with success, before any re-action, or, if we may use the expression, re-animation took place."—*New Medical and Physical Journal*, vol. x, p. 52.

Dr. Hutchison has given his own experience, and that of many others, in corroboration of what we have stated.

"It appears to me, however, that in all wounds from cannon-shot or splinters, or even those from musket balls, penetrating the joint of the knee, for instance, or in those attended with fracture and laceration of the principal nerves of the limb, &c. in which amputation may be deemed necessary on the first view of the case; that the sooner such a source of irritation is removed by a fresh incised wound, the greater will be the probability of recovery." P. 4.

The following is the testimony of Dr. Wright, now Physician to Haslar Hospital.

"I am no advocate for delay, when amputation is deemed necessary in recent wounds and accidents. I have always operated as soon as possible. I amputated during the whole of the Dutch action without the least intermission, until all were completed; and, in this battle, I had only the aid of two very young assistants.

"I have been informed, also, by some of my friends, who were in Lord Rodney's action, in the West Indies, commonly called the battle of the 12th of April, that in some of the ships, where the amputations were deferred until the action was over, the success was by no means such as to recommend the adoption of the plan in future practice." P. 16.

Dr. Hutchison, as well as ourselves, expresses his regret that Mr. Guthrie had not rendered himself acquainted with the naval practice on these occasions, before he made such a work about the discovery of what was known to every Surgeon's mate in the navy during the last fifty years.

*The Tourniquet* is recommended to be applied a hand's-breadth below the groin or axilla, whether the amputation be above or below the joints of the knee or elbow. He is in the habit of using a very small, hard pad, laid obliquely across the line of the artery, with the tourniquet nearly opposite, on the outside of the limb. Dr. H. cautions us against tying the vein with the artery, lest a branch of nerve should be included between them. He dissents from Mr. Guthrie's recommendation of cold water to the stump, because it gives additional pain, and causes a greater subsequent re-action. In respect to the line of junction on the face of a stump, Dr. H. is a strenuous advocate for the transverse instead of the perpendicular. He does not prefer this for the reasons assigned by Allanson, namely, that, in consequence of the more powerful action of the flexor muscles, the cicatrix may be drawn downwards, and the extremity of the bone covered with the old skin, instead of being opposite the cicatrix. The Doctor conceives that the perpendicular line of junction favours the formation of pus between the flaps, because the stump, while resting on its cushion, will have the lower part of the line forced open by the weight of the limb itself. This he illustrates by a figure which, at first sight, we took for a keg of ale or beer, resting on a bench, but bursting open from the strength of its contents, (a rare instance, by the bye, in these degenerate days !) It is meant, however, to represent a stump, with the lower angle of the wound opening asunder.

"This separation of the flaps, says he, however partial it may be, must necessarily form a cavity, and consequently a *nidus* for the secretion of matter."

Now we object to the probability of this opening of the line in the manner described by Dr. H. because, as he must well know, the *extremity* of the stump is never allowed to rest on a cushion at all, the support being always placed somewhat above the end of the amputated limb, so as to leave the face entirely free. When the operation is below the knee, we defy the most clumsy nurse to place the member in such a manner as to rest in the way described in the plate. But, granting that the separation did take place, and that a *nidus* for matter was the consequence; then, we say, that this nidus, or *nest*, is formed with the opening *downwards*, a kind of nest which few of our readers may be acquainted with; though, we well recollect how often we have gazed with astonishment at the instinctive sagacity of a bird in the eastern hemisphere, which we have seen construct its nest with the *door downwards*, and the fundus attached to the extremity of a slender and lofty branch. This was to prevent the unwelcome visits of lizards and other reptiles, in search either of eggs or young ones. But to return; we cannot see how this separation can be more liable to take place in the perpendicular than in the transverse line; and when it *does* take place, we think it is less disposed to afford a nidus for matter in the common, than Dr. Hutchison's mode of junction. We have just seen so many excellent stumps with perpendicular lines, returned from the Algerine conflict, that we are quite content as to the propriety of the present plan of operating.

Dr. H. recommends that the dressings be not removed before the fifth or sixth day, unless tension, inflammation, or unusual secretion of pus should demand an earlier interference. He offers, we think, a useful hint—that of replacing the old by fresh straps in the order of removal, and one by one. In the after treatment, Dr. H. justly observes, that the lancet has been too much dreaded when vascular re-action runs high, and he thinks that lives have been lost from this timidity. In the dysenteric complaints occasionally attending carious ulcers, the removal of the limb is the only measure which can be depended on. Dr. H. adds his suffrage to that of our colleague, Dr Johnson, in respect to the utility of calomel in large doses in chronic dysentery.

"I have found the exhibition of large doses of calomel in chronic dysentery, attended by the most happy results; and I embrace this opportunity of adding my testimony on this subject in corroboration of Mr. Johnson's opinions." P. 50.

Dr. H. here relates a very interesting case of secondary hæmorrhage, after an amputation for erysipelatous inflammation. An incision, in the direction of the artery was made, three inches in length, terminating on the face of the stump. The artery was taken up and the patient recovered. Our author thinks that the ulceration of the arterial coats, in this case, was occasioned by the nerve being torn from the side of the vessel, higher up than where the ligature was applied.

*Case.* R. F. a seaman, 32 years of age, had his leg dreadfully lacerated by the fall of a topmast, on board of H.M.S. Defence, in the Downs. He was immediately sent off for the hospital; but, owing to contrary winds and tide, he was three hours in the boat. During the first two hours, there was slight hæmorrhage, but the patient was perfectly tranquil and collected. At the expiration of this period, he was seized with a convulsive fit, which went off, and again recurred before he got on shore. By the time he arrived at the hospital, his jaws were closely locked. He was carried into the operation room, and soon after the amputation had commenced, he was seized with a third convulsion. Just as the stump was dressed he expired. The pad, which was a large one, had been placed over the femoral artery, and the tourniquet was screwed tight over that. Yet, during the division of the muscles, several gushes of arterial blood forced Dr. H to search for and tie the artery, before separating the limb. It was the profunda, which, in this case, went off from the external iliac, and by passing along in that space between the pad and the part of the web of the instrument which pressed upon the limb, the course of blood continued uninterrupted.

This case Dr. H. adduces in support of the propriety of amputating as soon after the accident as possible; it offers, indeed, an irresistible argument in favour of Baron Larrey's and our author's propositions. An interesting case of amputation of the foot at the tarso-metatarsal articulation is here related; but for which, we refer to the work itself, which, we hope, will be in the hands of most surgeons.

Dr. Hutchison corroborates, by two very interesting cases, the safety of amputating during the progress of gangrene, as maintained by Larrey, Guthrie, and Lawrence. We shall present the reader with some particulars of the second case. A seaman, of the middle age, was wounded through the knee-joint by a musket ball, off the coast of Holland.

"On the patient's admission into the hospital, four days after the injury, the leg was in a state of spreading gangrene, perfectly cold and livid round the ankle, and the lividness extending to about two or three inches above the wounded part. The patient complained of pain in the thigh, but more particularly in the region of the hip-joint: the thigh was therefore amputated within an hour after his presentation, close up to the trochanter; and he too recovered without any disposition to gangrene manifesting itself on the face of the stump." 75.

A long section follows on the treatment of erysipelatous inflammation by deep scarifications, principally reprinted from the Medico-Chirurgical Transactions, but enlarged by some additional observations on the medicinal part of the treatment. Dr. H. adheres to the term erys. phlegmon. rather than erys. œdematodes: we could wish him to quote a description of these two species from those systematic writers to whom he alludes; and if Dr. Bateman's work be taken as a standard, we are quite sure the erys. phlegmon. will not answer to the disease described by Dr. H. We recommend the paper, however, to the serious perusal of every practitioner; and we can vouch, in the most unqualified manner, for the truth of Dr. H's statements, and the great superiority of his plan of treatment.

*Popliteal Aneurism*.—So far back as the year 1800, Dr. H. tied the brachial arteries of two dogs, removing the ligatures immediately after their application, and found complete obliteration of the canal of the artery, in both instances, to be the result. Encouraged by these and other analogical reasonings and reflections, Dr. H. put in execution the plan of Mr. Travers, that of allowing the ligature to remain on the artery a few hours. On the 4th of November, 1813, Dominico Dalmeiro, a Venetian by birth, was admitted into Deal hospital, for the cure of popliteal aneurism of the right leg. The tumour was about the size of a small orange, and of a month's duration: its pulsations were strong, accompanied with considerable pain in the surrounding parts, extending to the leg beneath. No discoloration nor œdema of the limb; but the muscles were yielding and flaccid.—5th. A brisk cathartic was exhibited, and repeated on the 9th.—On the 10th he was bled to sixteen ounces.—11th. At a quarter before eleven an incision was made on the outer edge of the Sartorius muscle, and the theca of the femoral artery exposed. The artery was detached from the vein and nerve with great facility, and when a quarter of an inch was freed from attachments, a double ligature was placed under it. The upper ligature was firmly tied with a loop or slip knot, keeping the running end in reserve for its more easy disengagement. The lower ligature was then secured in a similar manner, leaving a space somewhat less than a quarter of an inch undivided. The wound was slightly closed, and the pulsation disappeared from the tumour, an undulatory motion only remaining. The ligatures were removed without the least disturbance to the vessel at the end of six hours. In less than half a minute after the removal of the ligatures, the artery became distended with blood, and the pulsation in the tumour was equally strong as before the operation! The vessel was therefore insulated a little more upwards and downwards, and two fresh ligatures passed under it, which were secured above and below the sites of the former ligatures. The ends of the ligatures were brought out in a direct line, the intervening portion of artery left undivided, and the lips of the wound approximated. It may here be remarked, that although the pulsations returned in the tumour after the removal of the first ligatures, "yet on placing our little fingers on the space between where the two ligatures had been applied, no pulsating action could be discovered either by my colleague or myself." p. 113. We refer Dr. Hutchison to the ingenious experiments of Dr. Parry, of Bath, or to the Review of them in the 7th Number of this Journal, for a satisfactory explanation of this phænomenon.

On the 5th day, when the dressings were removed, there were slight adhesions, with some good pus. On the 10th day also, appearances were favourable. On the 16th day, the undulatory motion had increased to a slight pulsation. On the 21st day, both ligatures came away, and every thing appeared to be doing well; but in the afternoon a hæmorrhage burst out from the inferior orifice of the artery, "*the blood did not flow* per saltum, *but in a full and uniform stream.*" The vessel was secured about an inch from the bleeding mouth. On the 5th of December, another hæmorrhage burst out from the upper portion of the artery; the extremity of which felt hard and thickened to the touch; the fundus of the wound was in a sloughing state. Amputation was now the dernier resort, and was performed high up. On the fifth day the dressings were removed, but the stump looked bad. Every thing that art and humanity could dictate was tried, but all in vain; he sank on the 21st of December.

This statement goes as far as any single fact can go, in proving that adhesion and obliteration do not follow the application of ligatures on the femoral artery of the human subject, when subsequently removed even after having been continued on for some hours.*

The examination of the limb discovered a curious phænomenon.

"Half way between the sac and the part of the artery that had been tied, two considerable branches *opened into the main trunk*, sufficiently large to have carried on the circulation through the old channel, and would therefore have prevented the cure of the disease for which the operation was, in the first instance, had recourse to. The gradual increase of pulsation in the tumour, after the operation, for a period of nearly four weeks, fully justifies the truth of this remark." p. 123.

Does Dr. H. mean to say, that a branch arising *from* the artery above the ligature opened *into* the artery below it; or that the blood became retrograde in these two considerable branches, which had, previously to the operation, been employed in carrying blood *from* the artery to the soft parts? If the former be the case, he corroborates the phænomenon mentioned by Dr. Parry as having happened in a sheep, and of which he has given a plate. We request Dr. H. to favour us with some information on

* The operation has been since performed twice in the London Hospitals with recovery; but the operation is objectionable, as seventy hours were required for the obliteration, and as secondary hæmorrhage ensued.

this very curious subject. It would have been well worth the trouble to have traced these two arteries carefully, to see whether they joined others by anastomosis, or were really superior branches that dipped back into the trunk below the ligatures to carry on the circulation.

Dr. Hutchison, in the details of this very interesting case, evinces much sensibility, candour, and good sense; characters indeed that are conspicuous throughout the work.

We shall transcribe the following case of wounded brachial artery, both as a caution to young gentlemen in the act of bleeding, and an instructive lesson in the event of their injuring the subjacent artery.

"Thomas Mellifont, a seaman of the York, seventy-four, on the 16th of March 1813, was attacked with incipient symptoms of pneumonia, for which the surgeon of the ship directed him to be bled. The assistant who performed the operation, owing to a pitch of the ship, then under sail, slit open the brachial artery more than a quarter of an inch in an oblique direction. The hæmorrhage was suppressed by a thick compress and roller until the 17th, when the patient was placed under my care.

"On the bandage and compress being removed, the blood gushed out in a *saltus*, and in larger volume than I supposed it possibly could have issued from the artery, if the vessel had been completely divided in a transverse direction. Pressure was therefore made upon the artery near the axilla, so as to stop the circulation in the vessel below, when I made an incision two inches in length over the punctures and in the direction of the vessel, until, by gentle scratches with the scalpel, the punctured part of the artery was reached; before we could clearly see the apertnre made into the artery, however, three small branches were tied, which bled freely and obscured our view. A probe was now passed through the opening into the canal of the artery upwards, which enabled us to divide the vessel completely at this part, to raise the divided upper end into which the probe had been introduced, and to detach it a little way from the great cardiac nerve and its cellular connexions, until we could lay hold of it with a pair of artery forceps, by which it was drawn downwards, and the artery firmly tied with a ligature, in the same manner as in an amputated stump. In detaching the lower divided end of the artery with the probe introduced, a few fibres of the tendon of the biceps were necessarily divided, and a ligature was in like manner applied here.

"The wound was cleared of blood, and the sides were brought together by slips of sticking-plaster. The ligatures came away in a few days, the wound very soon cicatrized, and left the patient in as full possession of the use of his arm, as if no such accident had occurred, and he was discharged to his duty." p. 129.

Although our analysis has extended beyond its relative bounds, we should have gone still further, did we not believe that the intrinsic merits of Dr. Hutchison's work, and the specimens which we have here adduced, will procure for it a very extensive perusal. To the Surgeon, the general Practitioner, and the Student, we recommend this little volume as a depositary of authentic and valuable facts, sensible observations, and ingenious suggestions.

